# Rapid and specific immunoPET imaging of Nectin-4 in gastric cancer and non-small cell lung cancer using [^64^Cu]Cu-NOTA-EV-F(ab’)_2_

**DOI:** 10.1007/s00259-025-07402-z

**Published:** 2025-06-21

**Authors:** Wenpeng Huang, Xinyao Sun, Xiaoyan Li, Jessica C. Hsu, Yongkang Qiu, Molly C. DeLuca, Jonathan W. Engle, Liming Li, Jun Lu, Tianyao Wang, Lei Kang, Weibo Cai

**Affiliations:** 1 Department of Nuclear Medicine, Peking University First Hospital, Beijing 100034, China; 2 Departments of Radiology and Medical Physics, University of Wisconsin–Madison, Madison, WI 53705, USA; 3 Department of Radiology, The First Affiliated Hospital of Zhengzhou University, Zhengzhou 450052, Henan Province, China; 4 Auckland Bioengineering Institute, University of Auckland, Auckland 1142, New Zealand; 5 Department of Food and Agriculture Technology, Yangtze Delta Region Institute of Tsinghua University, Jiaxing, China

**Keywords:** Cu-64, Nectin-4, ImmunoPET, F(ab’)_2_ fragments, Gastric cancer, Non-small cell lung cancers

## Abstract

**Purpose:**

This study aimed to develop and evaluate [^64^Cu]Cu-NOTA-EV-F(ab’)_2_ as a rapid and specific immunoPET imaging probe targeting Nectin-4 in gastric cancer (GC) and non-small cell lung cancer (NSCLC).

**Materials and methods:**

F(ab’)_2_ fragments were generated from enfortumab vedotin (EV) using IdeS protease and conjugated with *p*-SCN-Bn-NOTA for radiolabeling with ^64^CuCl_2_. The radiochemical yield was 85.40 ± 2.43% (*n* = 5). In vitro binding affinity and specificity were assessed *via* flow cytometry and cell uptake assays using Nectin-4-positive (NCI-N87, H1975) and Nectin-4-low (HGC-27, H520) cell lines. In vivo PET imaging and biodistribution studies were conducted in murine models of GC and NSCLC to evaluate tumor targeting efficiency and tracer pharmacokinetics.

**Results:**

[^64^Cu]Cu-NOTA-EV-F(ab’)_2_ demonstrated rapid tumor accumulation, with peak uptake observed at 4 h post-injection (10.23 ± 0.70%ID/g in NCI-N87 tumors, 3.03 ± 0.35%ID/g in HGC-27, 11.56 ± 1.12%ID/g in H1975, 2.77 ± 0.47%ID/g in H520). Compared to full-length EV, the tracer exhibited faster blood clearance and reduced off-target uptake. Blocking with excess EV-F(ab’)_2_ significantly reduced subsequent tumor uptake (6.27 ± 0.49%ID/g in NCI-N87, *P* = 0.0029; 5.23 ± 0.31%ID/g in H1975, *P* = 0.00074), confirming Nectin-4 specificity. Ex vivo biodistribution analysis supported high tumor retention consistent with in vivo imaging findings.

**Conclusions:**

[^64^Cu]Cu-NOTA-EV-F(ab’)_2_ offers rapid, specific, and high-contrast immunoPET imaging of Nectin-4-expressing tumors in GC and NSCLC models, highlighting its potential as a non-invasive diagnostic tool for Nectin-4-targeted cancer imaging.

## Introduction

Gastric cancer (GC) and non-small cell lung cancer (NSCLC) are among the most common and lethal malignancies globally. These cancers are difficult to detect early, have a high metastatic potential, and are generally associated with poor prognoses [1–4]. As a result, they pose a major public health challenge and place a burden on healthcare systems. Improving survival outcomes necessitates effective strategies for early diagnosis and ongoing disease monitoring to enable timely therapeutic intervention [5–7].

Nectin-4, a transmembrane protein overexpressed in various cancers, plays a pivotal role in tumorigenesis and metastasis [8–10]. The antibody-drug conjugate enfortumab vedotin (EV), which targets Nectin-4, is approved for treating advanced or metastatic urothelial carcinoma. However, its therapeutic potential in other cancer types, including GC and NSCLC, remains largely unexplored [11, 12].Despite the growing therapeutic relevance of Nectin-4–particularly, there remains a lack of clinically validated molecular imaging tools to noninvasively assess Nectin-4 expression.

ImmunoPET imaging represents a noninvasive approach to visualize the presence and spatial distribution of specific antigens in vivo [13, 14]. While full-length antibody-based imaging agents provide high specificity, they suffer from extended circulation times and slow accumulation in tumors, often requiring several days before optimal imaging window can be achieved [15, 16]. To address these limitations, F(ab’)_2_ fragments, lacking the immunoglobulin Fc region, have emerged as a promising alternative. Their faster blood clearance, reduced immunogenicity, and shorter biological half-life allow for lower dosages and earlier imaging [17].

In this study, we employed Ides protease to generate F(ab’)_2_ fragments derived from EV, with the goal of developing an optimized immunoPET tracer for Nectin-4 detection in GC and NSCLC models. This approach was hypothesized to reduce the waiting time to image while ensuring precise detection of the targeted antigen.

## Materials and methods

A comprehensive characterization of [^64^Cu]Cu-NOTA-EV and [^64^Cu]Cu-NOTA-EV-F(ab’)_2_ was performed using various analytical methods. Nectin-4 expression in human GC (NCI-N87, HGC-27) and NSCLC (H1975, H520) cell lines was assessed by flow cytometry (ThermoFisher Attune) and immunofluorescence imaging using an A1R confocal laser scanning microscope. Probe binding affinity and specificity were evaluated via cell uptake and binding assays.

Athymic Nude-Foxn1nu mice (Envigo) bearing NCI-N87, H1975, HGC-27, and H520 tumors were intravenously injected with [^64^Cu]Cu-NOTA-EV and [^64^Cu]Cu-NOTA-EV-F(ab’)_2_ (7.4–11.1 MBq/100 μL), followed by PET imaging at 1, 4, 12, 24, and 48 h post-injection. Ex vivo biodistribution studies were conducted after each PET scan. Tissue sections were further analyzed by anti-Nectin-4 immunostaining. Detailed experimental protocols are provided in the Supplementary material.

Quantitative data are expressed as mean ± standard deviation (SD). Statistical analyses were performed using GraphPad Prism version 8.0. Comparisons between two groups were conducted using Student’s t-tests, while comparisons across multiple groups were performed using one- or two-way ANOVA where appropriate. A *P*-value of < 0.05 was considered statistically significant.

## Results

### Preparation, radiolabeling, and characterization of EV-F(ab’)_2_

**Fig. 1:**
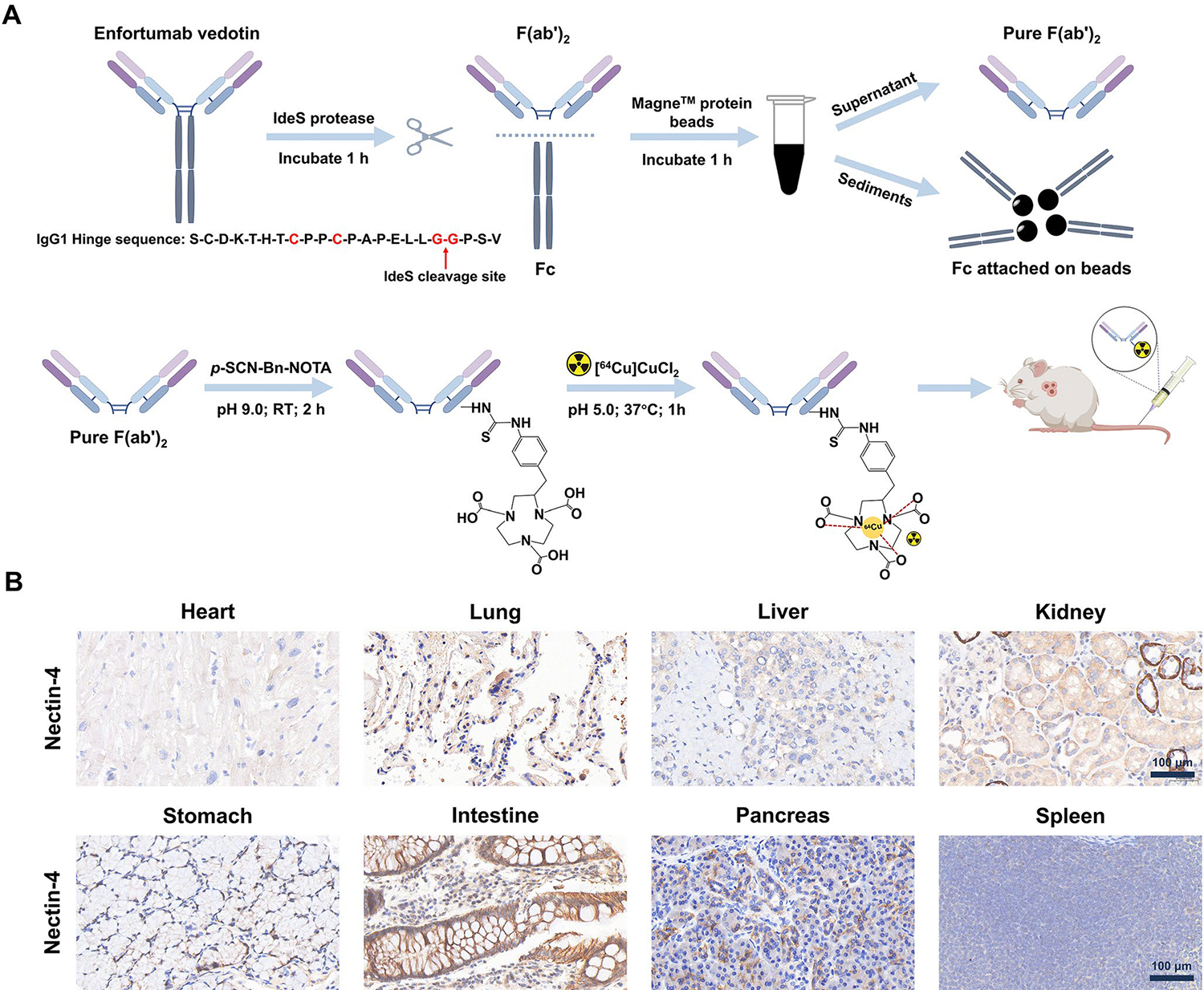
Preparation, radiolabeling, characterization of EV-F(ab’)_2_, and immunohistochemical analysis of Nectin-4 expression in human tissues. **(A)** Enzymatic digestion of EV mAb with IdeS protease, followed by purification with Magne^™^ Protein A beads and MagneHis^™^ Ni particles (top). The synthesis and radiolabeling steps for [^64^Cu] Cu-NOTA-EV-F(ab’)_2_ are shown (bottom). The final radiolabeled product was used for in vivo imaging studies. **(B)** Immunohistochemical staining of Nectin-4 expression in human tissues, including heart, lung, liver, kidney, stomach, intestine, pancreas, and spleen. Scale bar = 100 μm

As shown in Fig. 1A, EV was enzymatically digested using IdeS protease to generate the expected EV-F(ab’)_2_ fragments. These fragments were then purified using Magne^™^ Protein A beads and MagneHis^™^ Ni particles to effectively remove the Fc portion. Following purification, the EV-F(ab’)_2_ fragments were conjugated with *p*-SCN-Bn-NOTA at pH 9.0 and room temperature for 2 h. These mild reaction conditions preserved the integrity of their antigen-binding domains. Subsequent radiolabeling with ^64^CuCl_2_ at pH 5.0 and 37 °C for 1 h resulted in a high radiochemical yield (85.40 ± 2.43, *n* = 5), as verified by radio-ITLC (Figure S1, supporting information). The radiochemical purity exceeded 99% immediately after purification. Stability assessments showed that the probe maintained high radiochemical purity (> 98%) after 24 h incubation in both PBS and 5% human serum albumin at 37 °C, confirming excellent stability. Additionally, the measured specific activity of [^64^Cu]Cu-NOTA-EV-F(ab’)_2_ was approximately 0.36 ± 0.02 MBq/μg (*n* = 5).

SDS-PAGE analysis (Figure S2A, supporting information) confirmed the successful enzymatic cleavage of EV. The intact EV mAb (~ 150 kDa) was efficiently digested into F(ab’)_2_ fragments (~ 100 kDa). SDS-PAGE analysis showed that while EV appeared as a single band around 150 kDa, the EV-F(ab’)_2_ sample presented as a cluster of closely migrating bands around 100–130 kDa, which we attribute to mixed bands arising from microheterogeneity rather than incomplete enzymatic cleavage. HPLC analysis (Figure S2B, supporting information) further validated the integrity and purity of the fragments. Importantly, HPLC analysis confirmed a single dominant peak, supporting the overall integrity and purity of the EV-F(ab’)_2_ preparation. A single major peak was observed for both EV and EV-F(ab’)_2_ between 10 and 12 min. The retention time of EV-F(ab’)_2_ was delayed by approximately 1.5 min compared to intact EV, indicating successful fragmentation and separation.

### Immunohistochemical staining of Nectin-4 expression in human tissues

Immunohistochemical staining (Fig. 1B) was performed to assess the expression and distribution of Nectin-4 across different human organs. The results revealed strong Nectin-4 expression in the lung, kidney, and intestine, suggesting a significant presence of this protein in epithelial-rich tissues. In contrast, the heart, liver, stomach, pancreas, and spleen exhibited only weak to negligible expression, indicating that Nectin-4 has minimal or no involvement in these organs. These findings align with previously reported patterns of Nectin-4 expression and highlight the tissue-specific distribution of Nectin-4, which may have important implications for the development of Nectin-4-targeted diagnostics and therapeutics.

### Nectin-4 expression and cellular binding in GC and NSCLC cell lines

**Fig. 2:**
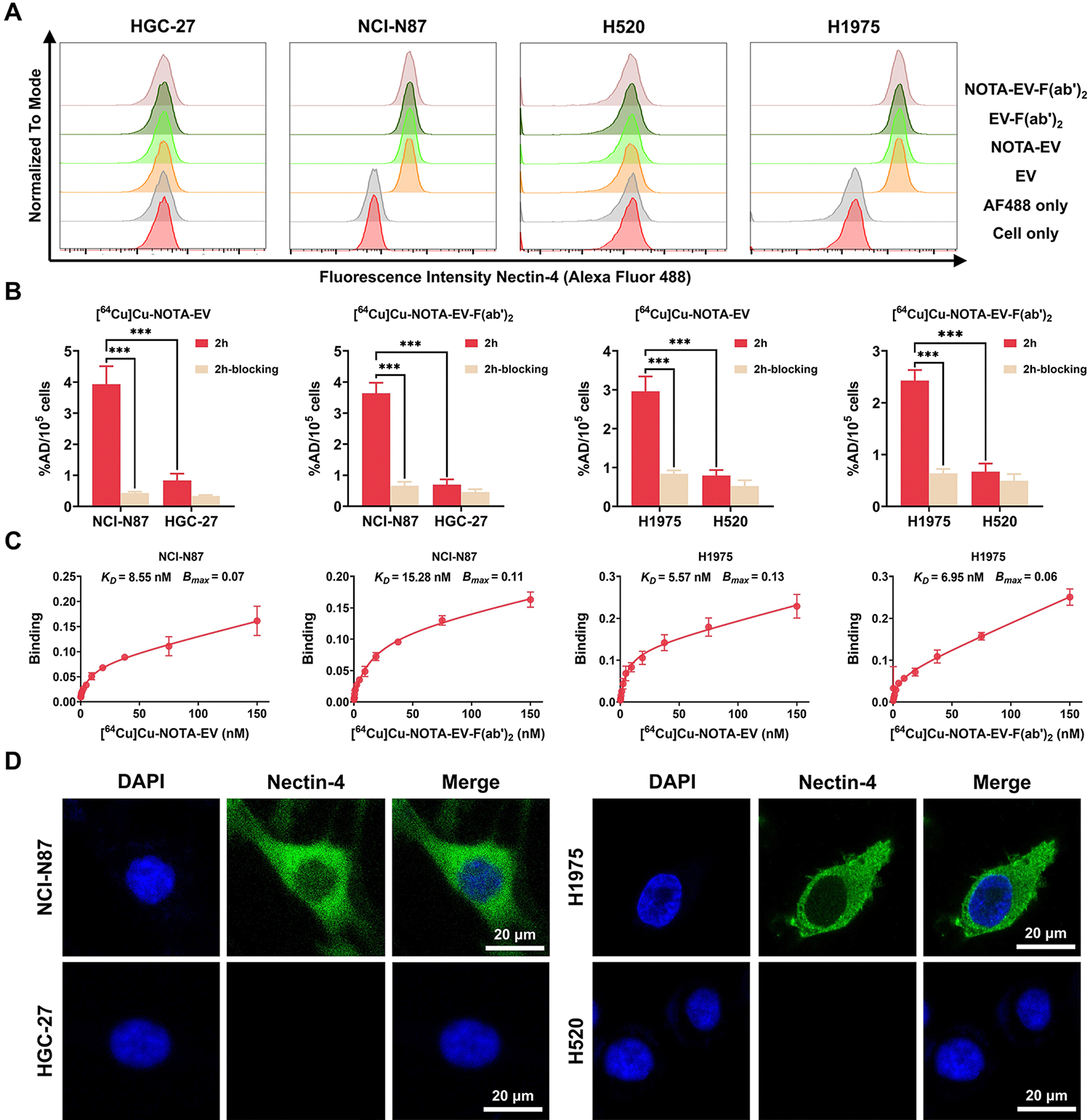
Nectin-4 expression and cellular binding of [^64^Cu]Cu-NOTA-EV and [^64^Cu]Cu-NOTA-EV-F(ab’)_2_ in cancer cell lines. (**A**) Flow cytometry analysis of Nectin-4 expression in human GC and NSCLC cell lines. Cells (NCI-N87, HGC-27, H1975, and H520) were incubated with Alexa Fluor 488-labeled probes (NOTA-EV, NOTA-EVF(ab’)_2_, EV, EV-F(ab’)_2_) or secondary antibody only (AF488) for 1 h on ice. Unstained controls were included for baseline fluorescence. Histogram plots display fluorescence intensity shifts indicative of Nectin-4 expression. NCI-N87 and H1975 show strong signal, while HGC-27 and H520 demonstrate low baseline fluorescence, consistent with Nectin-4 expression levels. (**B**) Cellular uptake assays of [^64^Cu] Cu-NOTA-EV and [^64^Cu]Cu-NOTA-EV-F(ab’)_2_ in NCI-N87, HGC-27, H1975, and H520 cells after 2 h of incubation, with and without blocking. Data are presented as mean ± SD (**P < 0.01, ***P < 0.001, *n* = 3). (**C**) Saturation binding curves of [^64^Cu]Cu-NOTA-EV and [^64^Cu]Cu-NOTA-EV-F(ab’)_2_ in NCI-N87 and H1975 cells (*n* = 3). (**D**) Immunofluorescence staining of Nectin-4 in NCI-N87, HGC-27, H1975, and H520 cells. Nuclei were counterstained with DAPI. Scale bar = 20 μm

Flow cytometry analysis (Fig. 2A) confirmed differential Nectin-4 expression across the two cancer types. Notably, NCI-N87 and H1975 exhibited higher fluorescence intensity, indicating elevated Nectin-4 expression compared to HGC-27 and H520. Based on these results, NCI-N87 and H1975 were designated as Nectin-4 high-expressing cell lines, while HGC-27 and H520 were categorized as low-expressing. Furthermore, fluorescence intensity was similar among cells incubated with Alexa Fluor 488-labeled NOTA-EV-F(ab’)_2_, EV-F(ab’)_2_, NOTA-EV, and EV—all of which showed higher signal than the Alexa Fluor 488-only and unstained control groups—indicating effective binding of EV to Nectin-4 protein.

A cell uptake assay was performed to assess the specificity of [^64^Cu]Cu-NOTA-EV and [^64^Cu]Cu-NOTA-EV-F(ab’)_2_ toward Nectin-4. As shown in Fig. 2B, both tracers demonstrated significant uptake in Nectin-4-expressing cells (NCI-N87 and H1975). In NCI-N87 cells, uptake was 3.93 ± 0.47%AD/10^5^ cells for [^64^Cu]Cu-NOTA-EV and 3.64 ± 0.28%AD/10^5^ cells for [^64^Cu]Cu-NOTA-EV-F(ab’)_2_. In H1975 cells, uptake values were 2.96 ± 0.31%AD/10^5^ cells and 2.43 ± 0.17%AD/10^5^ cells for [^64^Cu]Cu-NOTA-EV and [^64^Cu]Cu-NOTA-EV-F(ab’)_2_, respectively. No significant differences were observed between the full-length EV and EV-F(ab’)_2_ tracers. Furthermore, blocking significantly reduced tracer binding in all groups to levels comparable with Nectin-4 low-expressing cell lines (HGC-27 and H520), confirming the specificity of both tracers. For example, in NCI-N87 cells, uptake decreased from 3.93 ± 0.47 to 0.43 ± 0.04%AD/10^5^ cells after blocking, while uptake in HGC-27 cells was 0.84 ± 0.18%AD/10^5^ cells (*P* < 0.01). Direct statistical comparisons between [^64^Cu]Cu-NOTA-EV and [^64^Cu]Cu-NOTA-EV-F(ab’)_2_ demonstrated no significant difference in tracer uptake within NCI-N87 and H1975 cells (*P* > 0.05).

Saturation binding assays (Fig. 2C) further quantified binding affinity. The results showed that [^64^Cu]Cu-NOTA-EV displayed a higher binding affinity than [^64^Cu] Cu-NOTA-EV-F(ab’)_2_ in NCI-N87 cells, with *K*_*D*_ values of 8.55 nM and 15.28 nM, respectively. In H1975 cells, similar results were observed with *K*_*D*_ values of 5.57 nM for [^64^Cu] Cu-NOTA-EV compared to 6.95 nM for [^64^Cu]Cu-NOTA-EV-F(ab’)_2_. These findings suggest that the full-length EV construct may offer better targeting efficiency for Nectin-4. Regarding binding capacity (B_max_), [^64^Cu]Cu-NOTA-EV showed a higher capacity than [^64^Cu]Cu-NOTA-EV-F(ab’)_2_ in H1975 cells (0.13 vs. 0.06, respectively). However, [^64^Cu]Cu-NOTA-EV-F(ab’)_2_ showed a slightly higher binding capacity than [^64^Cu]Cu-NOTA-EV in NCI-N87 cells (0.11 vs. 0.07, respectively). As EV and its derivatives are known to internalize upon Nectin-4 binding, the cell-associated radioactivity measured after a 4 h incubation at 37 °C likely reflects both membrane-bound and internalized fractions. Therefore, the calculated *K*_*D*_ and B_max_ values represent apparent binding parameters under internalization-permissive conditions. Immunofluorescence imaging (Fig. 2D) corroborated flow cytometry findings, with strong Nectin-4 staining in NCI-N87 and H1975 cells and minimal fluorescence in HGC-27 and H520, confirming lower Nectin-4 expression in the latter.

### In vivo PET imaging and ex vivo biodistribution of [^64^Cu]Cu-NOTA-EV and[^64^Cu]Cu-NOTA-EV-F(ab’)_2_ in GC models

**Fig. 3:**
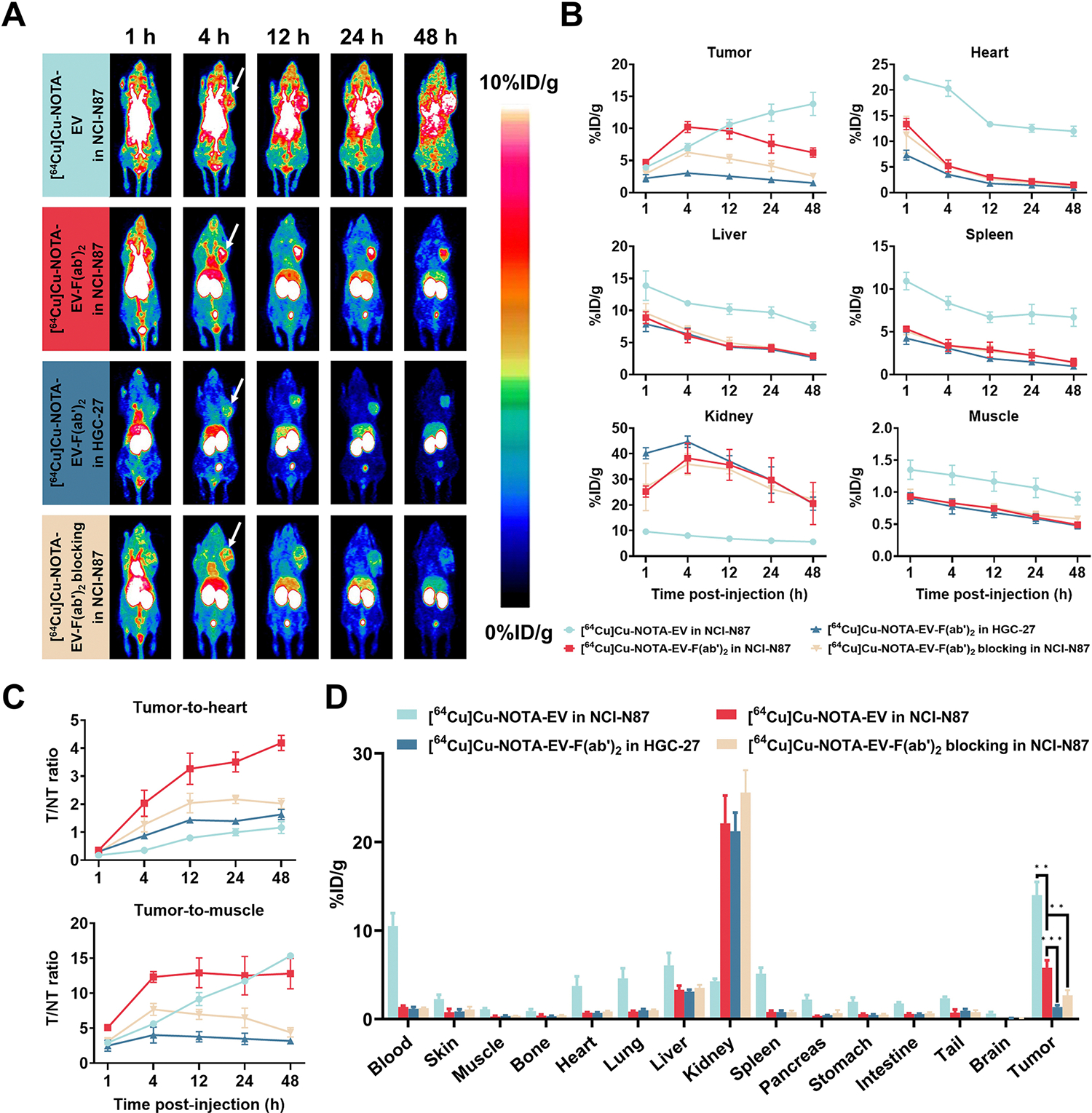
PET imaging and ex vivo biodistribution of [^64^Cu]Cu-NOTA-EV and [^64^Cu]Cu-NOTA-EV-F(ab’)_2_ in murine models of GC. (**A**) Representative PET images of mice bearing NCI-N87 or HGC-27 tumors following intravenous injection of [^64^Cu]Cu-NOTA-EV or [^64^Cu]Cu-NOTA-EV-F(ab’)_2_ at 1, 4, 12, 24, and 48 h p.i. Blocking studies were conducted by co-injecting an excess of unlabeled EV-F(ab’)_2_ in NCI-N87 tumor-bearing mice. (**B**) Quantitative ROI analysis of [^64^Cu]Cu-NOTA-EV and [^64^Cu]Cu-NOTA-EV-F(ab’)_2_ in tumors and major organs (heart, liver, spleen, kidney, and muscle) at 1, 4, 12, 24, and 48 h p.i. Cyan represents [^64^Cu]Cu-NOTA-EV in NCI-N87; Red represents [^64^Cu]Cu-NOTA-EV-F(ab’)_2_ in NCI-N87; Orange represents [^64^Cu]Cu-NOTA-EV-F(ab’)_2_ with blocking in NCI-N87; Blue represents [^64^Cu]Cu-NOTA-EV-F(ab’)_2_ in HGC-27. (**C**) Tumor-to-heart and tumor-to-muscle ratios of [^64^Cu]Cu-NOTA-EV and [^64^Cu] Cu-NOTA-EV-F(ab’)_2_ over time. (**D**) Ex vivo biodistribution of [^64^Cu] Cu-NOTA-EV and [^64^Cu]Cu-NOTA-EV-F(ab’)_2_ at 48 h p.i. Data are presented as mean ± SD (**P < 0.01, ***P < 0.001). *n* = 3/group

As shown in Fig. 3A–B, PET imaging and quantitative ROI analysis demonstrated distinct biodistribution patterns of [^64^Cu]Cu-NOTA-EV and [^64^Cu]Cu-NOTA-EV-F(ab’)_2_ in NCI-N87 and HGC-27 xenografts. [^64^Cu]Cu-NOTA-EV exhibited increasing tumor accumulation over time, reaching the highest uptake at 48 h p.i. (13.83 ± 1.89%ID/g). In contrast, [^64^Cu]Cu-NOTA-EV-F(ab’)_2_ displayed peak uptake at 4 h p.i. (10.23 ± 0.70%ID/g in NCI-N87; 3.03 ± 0.35%ID/g in HGC-27) and gradually cleared thereafter. Blocking with excess unlabeled EV-F(ab’)_2_ significantly reduced uptake in NCI-N87 tumors (6.27 ± 0.49%ID/g), confirming Nectin-4-specific binding of [^64^Cu]Cu-NOTA-EV-F(ab’)_2_ (*P =* 0.0029). In non-tumor organs, both tracers showed notable liver uptake at early time points, with [^64^Cu]Cu-NOTA-EV exhibiting sustained liver retention compared to [^64^Cu]Cu-NOTA-EV-F(ab’)_2_. Liver uptake of [^64^Cu]Cu-NOTA-EV gradually declined over time, while [^64^Cu]Cu-NOTA-EV-F(ab’)_2_ cleared more rapidly. A similar clearnace trend was observed in the spleen. In contrast, renal uptake was significantly higher for [^64^Cu]Cu-NOTA-EV-F(ab’)_2_, particularly at earlier time points, reflecting enhanced renal clearance due to its smaller molecular size. Renal retention of [^64^Cu]Cu-NOTA-EV-F(ab’)_2_ remained higher than that of [^64^Cu]Cu-NOTA-EV at 48 h p.i., consistent with the excretion pathway of antibody fragments. Heart uptake, representing blood circulation, was relatively low for both tracers and decreased over time. However, the faster decline in heart uptake for [^64^Cu]Cu-NOTA-EV-F(ab’)_2_ suggests more rapid systemic clearance compared to [^64^Cu]Cu-NOTA-EV, which exhibited prolonged circulation due to its larger size and intact Fc region. Muscle uptake remained consistently low across all time points, leading to high tumor-to-muscle contrast for both tracers. In Nectin-4-low expressing HGC-27 tumors, overall tumor uptake was lower compared to NCI-N87 xenografts, further supporting the specificity of [^64^Cu]Cu-NOTA-EV-F(ab’)_2_ to Nectin-4.

In Fig. 3C, the tumor-to-heart ratio of [^64^Cu]Cu-NOTA-EV-F(ab’)_2_ peaked at 48 h p.i., confirming efficient blood clearance and tumor-specific accumulation, whereas the tumor-to-muscle ratio showed an optimal tumor contrast at 12 h p.i. In NCI-N87 xenografts, [^64^Cu]Cu-NOTA-EV-F(ab’)_2_ achieved a tumor-to-heart ratio of 3.27 ± 0.55 at 12 h p.i., which was significantly higher than the negative control (1.43 ± 0.10, *P* = 0.00505) and blocking group (2.04 ± 0.28, *P* = 0.03223). [^64^Cu]Cu-NOTA-EV had a lower tumor-to-heart ratio at 12 h p.i. (0.80 ± 0.07, *P* = 0.00161 vs. [^64^Cu] Cu-NOTA-EV-F(ab’)_2_), indicating slower clearance from blood circulation. Similarly, the tumor-to-muscle ratio of [^64^Cu]Cu-NOTA-EV-F(ab’)_2_ in NCI-N87 tumors was 12.91 ± 2.10 at 12 h p.i., significantly higher than the negative control (3.77 ± 0.74, *P* = 0.00213) and blocking group (6.96 ± 0.69, *P* = 0.00995). At 12 h p.i., [^64^Cu]Cu-NOTA-EV had a tumor-to-muscle ratio of 9.15 ± 0.93, which was also significantly lower than [^64^Cu]Cu-NOTA-EV-F(ab’)_2_ (*P* = 0.04839).

Ex vivo biodistribution studies (Fig. 3D; Table S2, supporting information) confirmed significantly higher tumor uptake of [^64^Cu]Cu-NOTA-EV-F(ab’)_2_ in NCI-N87 and H1975 xenografts compared to control and blocking groups. At 48 h p.i. in NCI-N87 tumors, [^64^Cu]Cu-NOTA-EV-F(ab’)_2_ accumulation was 5.81 ± 0.69%ID/g, while uptake in the negative control and blocking groups was significantly lower (1.37 ± 0.16%ID/g, *P* = 0.00088; 2.68 ± 0.48%ID/g, *P* = 0.0061). Overall, these results demonstrate that both tracers showed specific Nectin-4 binding. In addition, ex vivo biodistribution analysis at 48 h post-injection revealed significantly higher tumor accumulation of [^64^Cu] Cu-NOTA-EV compared to [^64^Cu]Cu-NOTA-EV-F(ab’)_2_ in NCI-N87 xenografts (*P* = 0.0012), [^64^Cu]Cu-NOTA-EV exhibits superior tumor retention over time, while [^64^Cu] Cu-NOTA-EV-F(ab’)_2_ provides improved tumor-to-background contrast at earlier imaging time points.

### In vivo PET imaging and ex vivo biodistribution of [^64^Cu]Cu-NOTA-EV and [^64^Cu]Cu-NOTA-EV-F(ab’)_2_ in NSCLC models

**Fig. 4:**
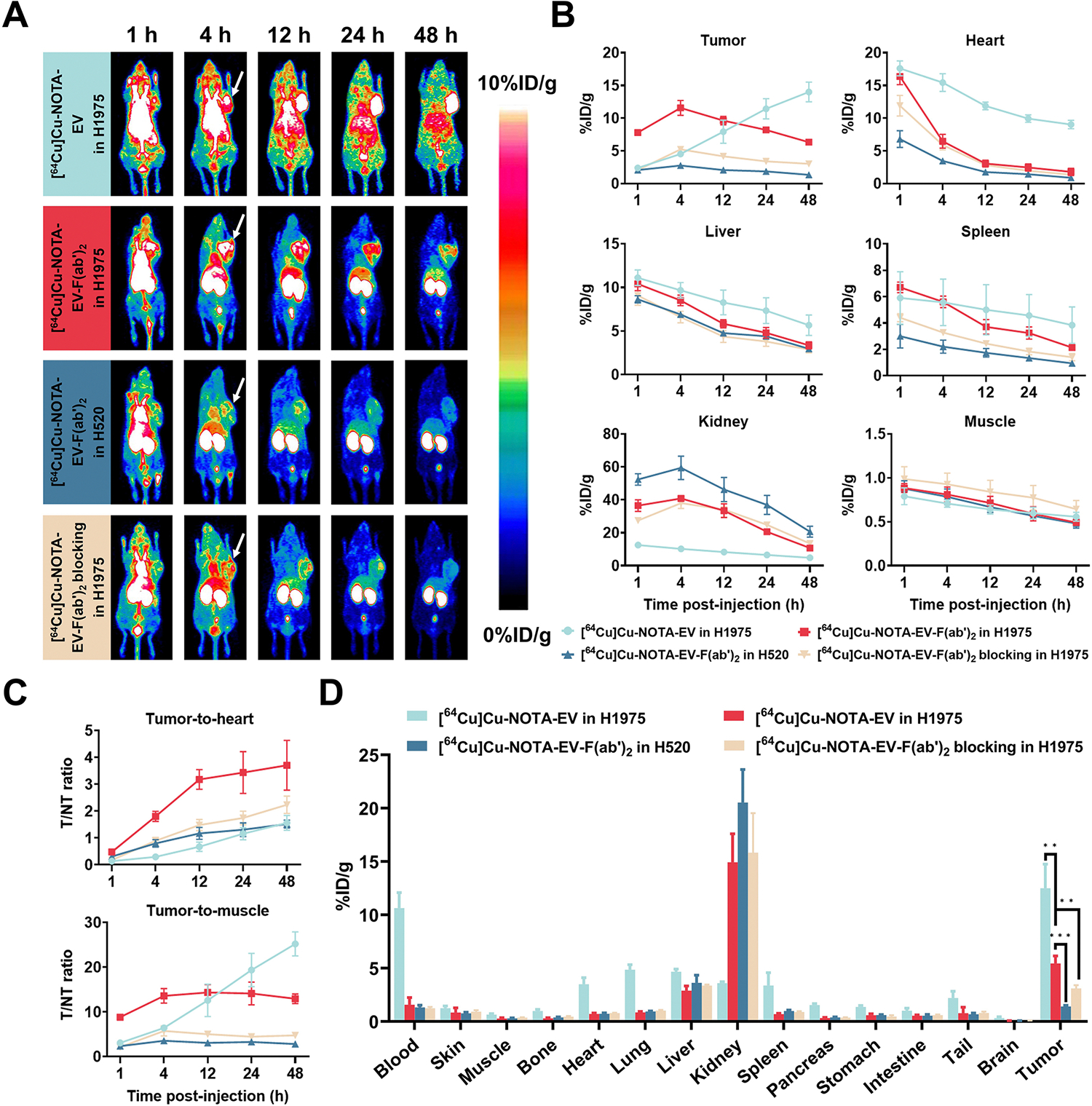
PET imaging and ex vivo biodistribution of [^64^Cu]Cu-NOTA-EV and [^64^Cu]Cu-NOTA-EV-F(ab’)_2_ in murine models of NSCLC. (**A**) Representative PET images of H1975 and H520 tumor-bearing mice at 1, 4, 12, 24, and 48 h p.i. following intravenous injection of [^64^Cu]Cu-NOTA-EV or [^64^Cu]Cu-NOTA-EV-F(ab’)_2_. Blocking studies were conducted by co-injecting an excess of unlabeled EV-F(ab’)_2_ in H1975 tumor-bearing mice. (**B**) Quantitative ROI analysis of [^64^Cu] Cu-NOTA-EV and [^64^Cu]Cu-NOTA-EV-F(ab’)_2_ in tumors and major organs (heart, liver, spleen, kidney, and muscle) at 1, 4, 12, 24, and 48 h p.i. Cyan represents [^64^Cu]Cu-NOTA-EV in H1975; Red represents [^64^Cu]Cu-NOTA-EV-F(ab’)_2_ in H1975; Orange represents [^64^Cu]Cu-NOTA-EV-F(ab’)_2_ with blocking in H1975; Blue represents [^64^Cu]Cu-NOTA-EV-F(ab’)_2_ in H520. (**C**) Tumor-to-heart and tumor-to-muscle ratios of [^64^Cu]Cu-NOTA-EV and [^64^Cu]Cu-NOTA-EV-F(ab’)_2_ over time. (**D**) Ex vivo biodistribution of [^64^Cu]Cu-NOTA-EV and [^64^Cu]Cu-NOTA-EV-F(ab’)_2_ at 48 h p.i. Data are presented as mean ± SD (**P < 0.01, ***P < 0.001). *n* = 3/group

The tumor-targeting efficiency and specificity of both [^64^Cu] Cu-NOTA-EV and [^64^Cu]Cu-NOTA-EV-F(ab’)_2_ were also evaluated in NSCLC xenografts. As shown in Fig. 4A–B, PET imaging demonstrated time-dependent tumor accumulation of [^64^Cu]Cu-NOTA-EV in H1975 xenografts, with uptake increasing over time and reaching a peak of 14.00 ± 1.22%ID/g at 48 h p.i. In contrast, [^64^Cu]Cu-NOTA-EV-F(ab’)_2_ exhibited peak tumor uptake of 11.56 ± 1.12%ID/g at 4 h p.i., followed by gradual clearance. At the same time point, blocking experiments significantly reduced tumor uptake (5.23 ± 0.31%ID/g, *P* = 0.00074), while uptake in the H520 negative group was even lower (2.77 ± 0.47%ID/g, *P* = 0.00024), confirming the specificity of [^64^Cu]Cu-NOTA-EV-F(ab’)_2_ for Nectin-4 targeting. A similar biodistribution pattern was observed in non-tumor organs between the GC and NSCLC mouse models.

Tumor-to-background ratios are shown in Fig. 4C. At 12 h p.i., [^64^Cu]Cu-NOTA-EV-F(ab’)_2_ achieved a significantly higher tumor-to-heart ratio of 3.18 ± 0.37 compared to the negative control (1.17 ± 0.22, *P* = 0.00124) and blocking group (1.48 ± 0.21, *P* = 0.00219). Meanwhile, [^64^Cu] Cu-NOTA-EV showed a much lower tumor-to-heart ratio (0.67 ± 0.17, *P* = 0.00043), indicating that the F(ab’)_2_ variant offers superior tumor contrast. Similarly, the tumor-to-muscle ratio for [^64^Cu]Cu-NOTA-EV-F(ab’)_2_ reached 14.36 ± 1.75 at 12 h p.i., significantly higher than the H520 negative control (3.08 ± 0.34, *P* = 0.00040) and blocking group (5.02 ± 0.68, *P* = 0.00102). In contrast, [^64^Cu]Cu-NOTA-EV had a tumor-to-muscle ratio of 12.59 ± 3.62, which was comparable to that of [^64^Cu]Cu-NOTA-EV-F(ab’)_2_.

Ex vivo biodistribution at 48 h p.i. (Fig. 4D; Table S3, supporting information) further validated the imaging findings. [^64^Cu]Cu-NOTA-EV-F(ab’)_2_ demonstrated significantly higher tumor retention in the H1975 Nectin-4-positive group (5.45 ± 0.57%ID/g) compared to the H520 negative control (1.41 ± 0.09%ID/g, *P* = 0.0006) and the blocking group (3.10 ± 0.26%ID/g, *P* = 0.0061). In addition, ex vivo biodistribution analysis at 48 h post-injection revealed significantly higher tumor accumulation of [^64^Cu] Cu-NOTA-EV compared to [^64^Cu]Cu-NOTA-EV-F(ab’)_2_ in H1975 xenografts (*P* = 0.0066). These results confirm the specificity of [^64^Cu]Cu-NOTA-EV-F(ab’)_2_ and highlight its potential for NSCLC imaging.

### Immunohistochemical and H&E staining

**Fig. 5:**
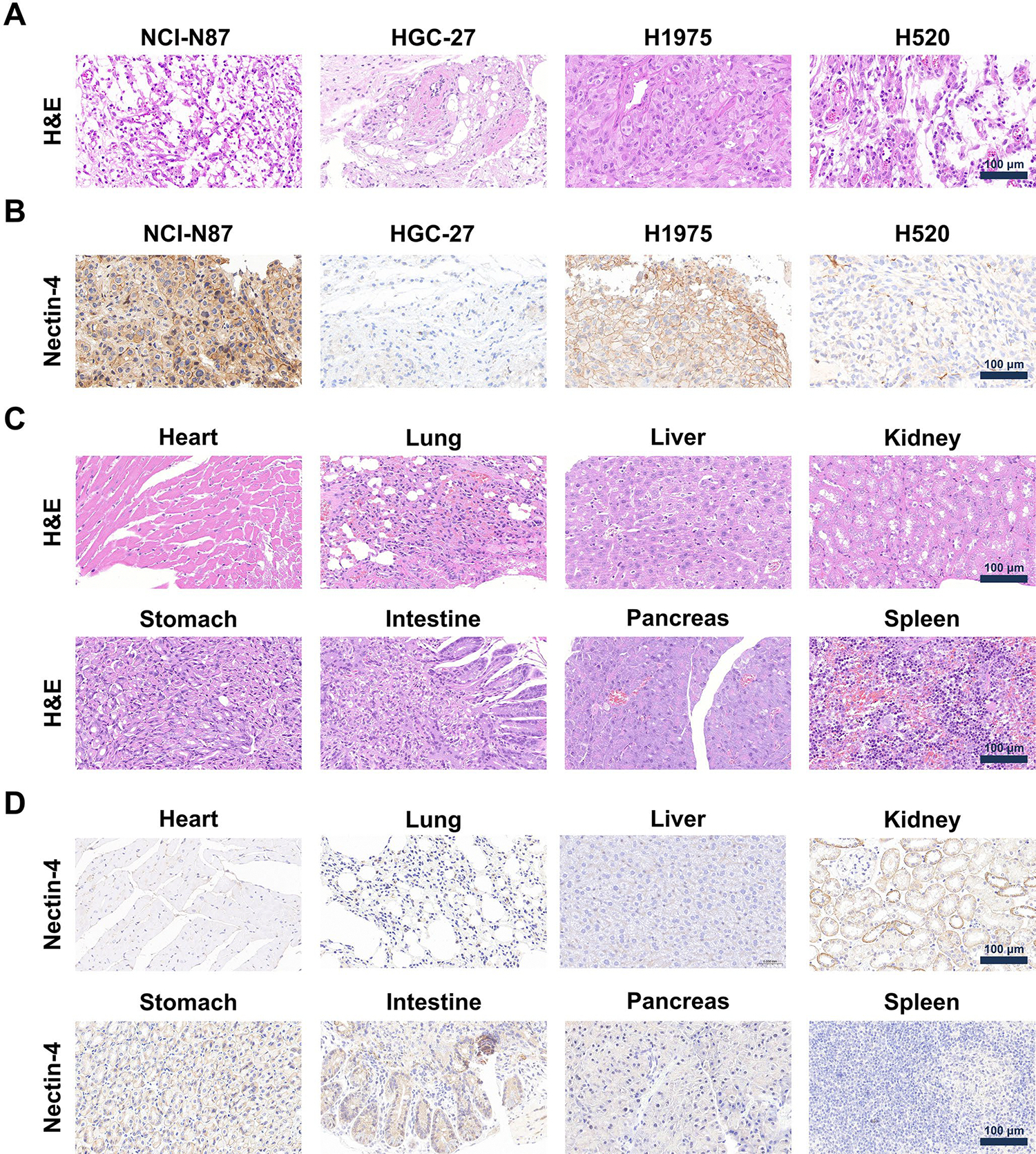
H&E and immunohistochemical analysis of Nectin-4 expression in murine tumor and normal tissues. (**A**) H&E staining of NCI-N87, HGC-27, H1975, and H520 tumors. (**B**) Immunohistochemical staining for Nectin-4 expression in NCI-N87, HGC-27, H1975, and H520 tumors. (**C**) H&E staining of heart, lung, liver, kidney, stomach, intestine, pancreas, and spleen. (**D**) Immunohistochemical staining for Nectin-4 expression in heart, lung, liver, kidney, stomach, intestine, pancreas, and spleen. Scale bar: 100 μm

H&E staining confirmed the histological characteristics of NCI-N87, HGC-27, H1975, and H520 tumors (Fig. 5A). IHC staining revealed high Nectin-4 expression in NCI-N87 and H1975 tumors, while HGC-27 and H520 exhibited minimal staining (Fig. 5B), supporting the selective expression of Nectin-4 in these tumor tissues. In normal organs (Fig. 5C), H&E staining results indicated no significant pathological changes in the heart, lung, liver, kidney, stomach, intestine, pancreas, and spleen, suggesting no observable toxicity associated with the administration of [^64^Cu] Cu-NOTA-EV-F(ab’)_2_. In normal tissues, IHC staining (Fig. 5D) demonstrated varying levels of Nectin-4 expression: strong staining in the lung, kidney, and intestine; weak staining in the heart, liver, stomach, and pancreas; and negligible staining in the spleen. These findings confirm that the safety of [^64^Cu]Cu-NOTA-EV-F(ab’)_2_ in both GC and NSCLC models, reinforcing its potential for further clinical development of Nectin-4 targeting in cancer diagnosis and therapy.

### Immunofluorescence staining

**Fig. 6:**
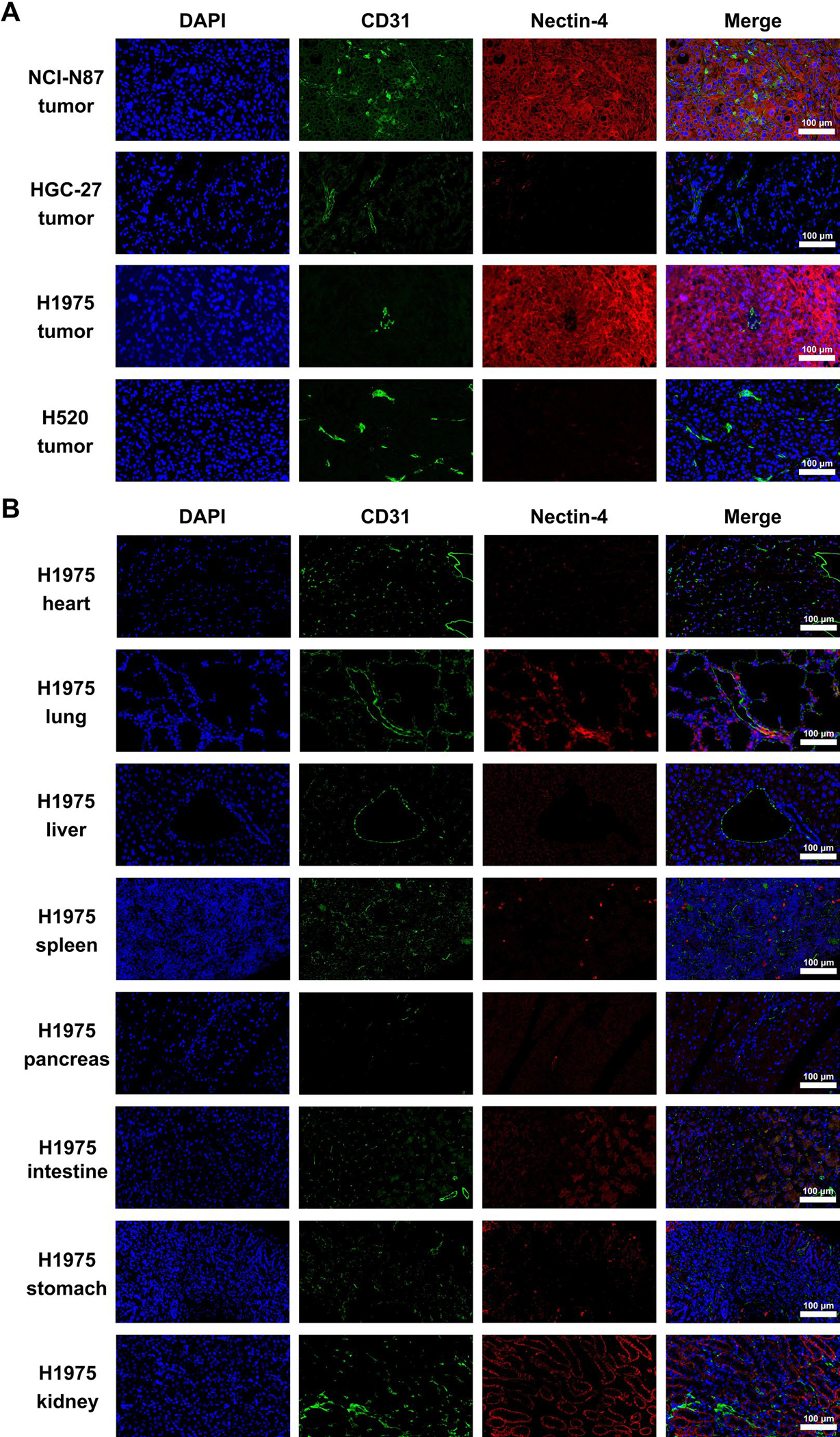
Immunofluorescence analysis of Nectin-4 expression and vascularization in tumors and normal tissues. (**A**) Representative immunofluorescence staining of tumor tissues (NCI-N87, HGC-27, H1975, and H520) for Nectin-4 (red) and CD31 (green). Nucleus staining with DAPI (blue) is shown for reference. (**B**) Immunofluorescence staining of major normal organs from H1975 tumor-bearing mice, including heart, lung, liver, spleen, pancreas, stomach, intestine, and kidney. Scale bar: 100 μm

Immunofluorescence staining was performed to evaluate Nectin-4 expression and vascularization in tumors and major normal organs. In tumor tissues (Fig. 6A), strong Nectin-4 staining was observed in NCI-N87 and H1975 tumors, consistent with the immunohistochemistry results, while HGC-27 and H520 tumors displayed minimal expression. CD31 staining revealed varying levels of vascularization, with NCI-N87 and H520 tumors showing a well-developed vascular network. In normal tissues (Fig. 6B), Nectin-4 expression was prominent in the lung, kidney, and intestine, with weak staining in the heart and minimal or no expression in the liver, spleen, pancreas, and stomach. CD31 staining highlighted the vascular architecture of each organ, with particularly strong endothelial signals in the heart, lung, and kidney. The selective expression of Nectin-4 in tumor tissues and limited distribution in normal organs further support the potential of [^64^Cu]Cu-NOTA-EV-F(ab’)_2_ as a promising tracer for Nectin-4 targeting.

### Radiation dosimetry extrapolation

Table 1 summarizes the estimated radiation doses deposited in human organs from [^64^Cu]Cu-NOTA-EV-F(ab’)_2_ based on biodistribution data. Likewise, Supplementary Table 1 contains the radiation dose estimates for [^64^Cu]Cu-NOTA-EV. For an adult woman, the calculated systemic effective dose was 0.0353 mSv/MBq, which is within the acceptable range for standard nuclear medicine studies, and provides a rational basis for planning future clinical investigations, pending validation through first-in-human studies.

## Discussion

ImmunoPET is an advanced molecular ima ging technique that enables noninvasive visualization of target antigens with high specificity, strong affinity, and enhanced sensitivity [18, 19]. Recent progress has emphasized the enzymatic breakdown of monoclonal antibodies to generate F(ab’)_2_ fragments, which have demonstrated therapeutic benefits [20, 21]. EV, an approved antibody-drug conjugate targeting Nectin-4, is currently used in patients with locally advanced or metastatic urothelial carcinoma. However, its diagnostic or therapeutic utility in other malignancies remains underexplored. In this study, we aim to explore the use of EV-derived antibody fragments in immunoPET imaging to evaluate Nectin-4 expression in models of GC and NSCLC.

Nectin-4 is primarily expressed in the placenta, skin, lungs, and urinary system, with minimal expression in most normal adult tissues, making it an attractive biomarker for cancer diagnosis and monitoring. In earlier studies, Shao et al. [14] developed a monoclonal antibody targeting Nectin-4 (mAbNectin-4) for both imaging and therapeutic purposes. Using [^99m^Tc]Tc-HYNIC-mAbNectin-4 for immuno-SPECT imaging, they observed significantly higher tumor uptake in MDA-MB-468 xenografts compared to MCF-7 xenografts (15.32 ± 1.04%ID/g vs. 3.02 ± 0.20%ID/g, *P* < 0.001). Additionally, the mAbNectin-4-ICG conjugate enabled efficient photothermal therapy, achieving complete tumor ablation with localized heating above 55 °C within five minutes (*P* < 0.0001). Compared with [^99m^Tc]Tc-HYNIC-mAbNectin-4, which may deliver higher radiation doses due to prolonged blood circulation and organ accumulation, our intermediate-sized F(ab’)_2_ tracer labeled with ^64^Cu significantly reduces radiation exposure while preserving effective targeting. The calculated effective dose for [^64^Cu]Cu-NOTA-EV-F(ab’)_2_ (0.0353 mSv/MBq) is well within acceptable clinical limits, potentially advantageous over longer-lived isotopes and larger antibodies that necessitate higher administered activities and extended imaging intervals. In addition, our fragment-based [^64^Cu]Cu-NOTA-EV-F(ab’)_2_ tracer strikes an effective balance by providing rapid tumor accumulation with peak contrast achievable at an intermediate imaging window (4–12 h p.i.). Although direct in vivo metabolite analysis was not performed, the absence of nonspecific tracer accumulation in clearance-associated organs (e.g., liver, bone) and the sustained high-contrast PET signals in tumors suggest that [^64^Cu]Cu-NOTA-EV-F(ab’)_2_ maintains high in vivo stability, consistent with its in vitro stability profile. The low-level uptake in HGC-27 and H520 tumors, despite negative results in histological and immunofluorescence analyses, may be attributed to baseline physiological perfusion and nonspecific tracer retention.

Duan et al. [22] explored another Nectin-4-targeting agent, the bicyclic peptide N188 labeled with ^68^Ga, for immuno-PET imaging. Results showed that SW780 tumors had significantly higher uptake (SUV_max_ 1.72 ± 0.15) compared to T24 tumors (SUV_max_ 0.56 ± 0.07, *P* < 0.001). Co-administration with unlabeled N188 led to a marked reduction (65%) in SW780 tumor uptake (*P* < 0.01), confirming the probe’s specificity. In a clinical study involving healthy volunteers and patients with advanced urothelial carcinoma, the SUV_max_ of [^68^Ga]Ga-N188 was notably higher in individuals with Nectin-4-positive bladder lesions (2.34 ± 0.21) and hepatic metastases (4.52 ± 0.33) compared to Nectin-4-negative individuals (*P* < 0.01). [^68^Ga]Ga-N188 generally achieves rapid tumor uptake but can suffer from rapid washout, potentially limiting sustained contrast. Our [^64^Cu]Cu-NOTA-EV-F(ab’)_2_ offers high initial tumor uptake coupled with rapid systemic clearance, achieving favorable and sustained tumor-to-background contrast as demonstrated quantitatively (e.g., tumor-to-muscle ratios of ~ 12.9 at 12 h p.i.).

Despite these advances, many antibody-based imaging agents suffer from suboptimal pharmacokinetics and prolonged circulation times, often requiring imaging windows up to three weeks post-injection. As a result, long-lived radionuclides, such as ^89^Zr and ^124^I are commonly used. However, they increase radiation exposure and delay image acquisition, limiting their clinical applicability. In contrast, small molecular imaging tracers have emerged as more suitable alternatives, enabling same-day imaging with reduced radiation burden.

Fab and F(ab’)_2_ fragments are advantageous when paired with short-lived positron-emitting isotopes like ^64^Cu and ^68^Ga, due to their faster clearance and more favorable pharmacokinetics. For example, CD20, predominantly expressed on B cells, is a key therapeutic target in non-Hodgkin lymphoma and chronic lymphocytic leukemia [23]. Suman et al. [24] developed [^68^Ga]Ga-labeled rituximab Fab and F(ab’)_2_ fragments to improve imaging specificity, though evaluation was limited to in vitro and non-tumor-bearing mouse models. Kang et al. [16] used IdeS to generate F(ab’)_2_ fragments from obinutuzumab, which were labeled with ^64^Cu for immunoPET imaging of CD20 in lymphoma xenografts. At 12 h p.i., [^64^Cu]Cu-NOTA-obinutuzumab-F(ab’)_2_ demonstrated significant tumor uptake (9.08 ± 1.67%ID/g in Ramos tumors), far surpassing uptake in negative control groups and highlighting its potential for noninvasive CD20 assessment in lymphomas. Additionally, CD103, a marker primarily located on intraepithelial lymphocytes and subsets of regulatory T cells, plays a role in mucosal immunity and tumor immunosurveillance [25]. Fan et al. [26] radiolabled anti-CD103 Fab fragments with ^89^Zr and ^68^Ga, finding that [^89^Zr]Zr-hCD103.Fab01A showed high tumor uptake and favorable imaging contrast at 24 h. However, the short half-life of ^68^Ga limited the imaging utility of its corresponding conjugate.

One limitation of our study is the notably high and persistent renal uptake observed following [^64^Cu]Cu-NOTA-EV-F(ab’)_2_ injection [27]. This issue arises mainly from the proteolytic degradation of radiolabeled fragments within lysosomes, thus producing radiolabeled catabolites in renal tubular cells following glomerular filtration [28]. Minimizing renal retention is crucial for improving the clinical utility of these imaging agents, and future studies should focus on overcoming this hurdle. Although our immunohistochemical and immunofluorescent analyses demonstrate substantial Nectin-4 expression in normal lung tissues, our in vivo PET imaging and ex vivo biodistribution results indicate clear and significant differentiation between tumor tissue (NSCLC xenografts) and adjacent normal lung tissues, may result from several factors: higher density and accessibility of Nectin-4 antigen; altered perfusion and vascular permeability; fragment-based pharmacokinetics. Future studies employing orthotopic and metastatic models are warranted to more rigorously evaluate the clinical translational potential of our Nectin-4-targeted probes. Further evaluations, including quantitative assessment of contrast ratios in patient-derived tumors versus normal lung tissues, will be essential to conclusively determine the appropriateness of Nectin-4-targeted imaging in clinical NSCLC diagnosis and monitoring.

We generated F(ab’)_2_ fragments through enzymatic digestion of intact IgG using IdeS protease, achieving high purity. However, ensuring consistent large-scale production under GMP-compliant conditions remains a challenge. Although [^64^Cu]Cu-NOTA-EV-F(ab’)_2_ displayed a modestly lower binding affinity in vitro, its favorable in vivo pharmacokinetics led to significantly improved tumor-to-muscle and tumor-to-heart ratios at early imaging time points compared to [^64^Cu]Cu-NOTA-EV. These findings suggest that the slightly lower affinity is effectively offset by rapid clearance and reduced background uptake, which are critical for achieving optimal PET imaging contrast and diagnostic utility. Additionally, exploring other antibody fragment types, such as diabodies, single-chain variable fragments (scFvs), Fab fragments, and nanobodies, may provide improved pharmacokinetics and better tumor-to-background ratios. Their potential for Nectin-4-targeted imaging warrants continued investigation.

## Conclusion

Our findings demonstrate that [^64^Cu]Cu-NOTA-EV-F(ab’)_2_ efficiently targets and accumulates in tumor tissues, exhibiting rapid uptake and sustained retention. These properties allow for precise and noninvasive imaging of Nectin-4 expression, supporting its potential as a valuable tool for stratifying patients and tracking tumor progression in GC and NSCLC.

## Supplementary Material

SI

**Supplementary Information** The online version contains supplementary material available at https://doi.org/10.1007/s00259-025-07402-z.

## Figures and Tables

**Table 1:** Radiation dosimetry Estimation of [^64^Cu]Cu-NOTA-EV-F(ab’)_2_ in human organs

Target Organ	mSv/MBq

Adrenals	3.77E-04
Brain	6.77E-05
Breasts	3.48E-03
Esophagus	1.28E-03
Eyes	0.00E00
Gallbladder wall	3.42E-04
Left colon	1.79E-03
Small intestine	4.08E-04
Stomach wall	5.27E-03
Right colon	1.75E-03
Rectum	8.24E-04
Heart wall	5.24E-04
Kidneys	1.86E-03
Liver	1.35E-03
Lungs	4.17E-03
Ovaries	1.44E-03
Pancreas	3.64E-04
Salivary glands	3.00E-04
Red marrow	6.11E-03
Osteogenic cells	3.67E-04
Spleen	2.71E-04
Thymus	3.12E-04
Thyroid	1.24E-03
Urinary bladder wall	1.27E-03
Uterus	1.65E-04
Effective dose	3.53E-02

Estimated absorbed radiation doses (mSv/MBq) for an adult female were extrapolated from murine biodistribution data using OLINDA/EXM software based on monoexponential fitting of time-activity curves. *n* = 3 per group, mean ± SD

## Data Availability

The datasets generated during and/or analyzed during the current study are available from the corresponding author on reasonable request.

## References

[1] Wen J, Cui W, Yin X, Chen Y, Liu A, Wang Q, Application and future prospects of bispecific antibodies in the treatment of non-small cell lung cancer. Cancer Biol Med. 2025;j.issn.2095-3941.2024.0470. 10.20892/j.issn.2095-3941.2024.0470 PMC12032835 40192238

[2] de Jong D, Das JP, Ma H, Pailey Valiplackal J, Prendergast C, Roa T, Novel targets, novel treatments: the changing landscape of non-small cell lung cancer. Cancers (Basel). 2023;15:2855. 37345192 10.3390/cancers15102855 PMC10216085

[3] He Q, Liu X, Jiang L, Liu P, Xuan W, Wang Y, First-line treatments for KRAS-mutant non-small cell lung cancer: current state and future perspectives. Cancer Biol Ther. 2025;26:2441499. 39681355 10.1080/15384047.2024.2441499 PMC11651285

[4] Ruan D, Zhao L, Cai J, Xu W, Sun L, Li J, Evaluation of FAPI PET imaging in gastric cancer: a systematic review and meta-analysis. Theranostics. 2023;13:4694–710. 37649615 10.7150/thno.88335 PMC10465231

[5] Huang W, Li L, Liang Y, Yang Q, Mixdorf JC, Engle JW, ImmunoPET imaging of Nectin4 expression in gastric and bladder cancer using [64Cu]Cu-NOTA-Padcev. Mol Pharm. 2025;22:3468–78. 40338113 10.1021/acs.molpharmaceut.5c00469 PMC12261917

[6] Huang W, Wang T, Chao F, Yang Q, Mixdorf JC, Li L, ImmunoPET imaging of Trop2 expression in bladder cancer using [64Cu]Cu-NOTA-Trodelvy. Mol Pharm. 2025;22:2266–75. 40059341 10.1021/acs.molpharmaceut.5c00069 PMC11978314

[7] Huang W, Li L, Zhou Y, Yang Q, Mixdorf JC, Barnhart TE, Preclinical evaluation of zirconium-89 labeled anti-Trop2 antibody-drug conjugate (Trodelvy) for imaging in gastric cancer and triple-negative breast cancer. Eur J Nucl Med Mol Imaging. 2025;52:2369–83. 39878898 10.1007/s00259-025-07106-4 PMC12119234

[8] Samanta D, Almo SC. Nectin family of cell-adhesion molecules: structural and molecular aspects of function and specificity. Cell Mol Life Sci. 2015;72:645–58. 25326769 10.1007/s00018-014-1763-4 PMC11113404

[9] Mendelsohn CL, Wimmer E, Racaniello VR. Cellular receptor for poliovirus: molecular cloning, nucleotide sequence, and expression of a new member of the immunoglobulin superfamily. Cell. 1989;56:855–65. 2538245 10.1016/0092-8674(89)90690-9

[10] Delpeut S, Noyce RS, Richardson CD. The tumor-associated marker, PVRL4 (nectin-4), is the epithelial receptor for morbilliviruses. Viruses. 2014;6:2268–86. 24892636 10.3390/v6062268 PMC4074928

[11] Khosravanian MJ, Mirzaei Y, Mer AH, Keyhani-Khankahdani M, Abdinia FS, Misamogooe F, Nectin-4-directed antibody-drug conjugates (ADCs): spotlight on preclinical and clinical evidence. Life Sci. 2024;352:122910. 39002610 10.1016/j.lfs.2024.122910

[12] Wong JL, Rosenberg JE. Targeting nectin-4 by antibody-drug conjugates for the treatment of urothelial carcinoma. Expert Opin Biol Ther. 2021;21:863–73. 34030536 10.1080/14712598.2021.1929168 PMC8224177

[13] Huang W, Zhang Y, Xiao X, Yang Q, Mixdorf JC, Sun X, ImmunoPET imaging of Trop2 expression in triple-negative breast cancer using [64Cu]Cu-NOTA-Trodelvy-F(ab’)2. Eur J Nucl Med Mol Imaging. 2025. 10.1007/s00259-025-07167-5 PMC12221780 39994021

[14] Shao F, Pan Z, Long Y, Zhu Z, Wang K, Ji H, Nectin-4-targeted immunoSPECT/CT imaging and photothermal therapy of triple-negative breast cancer. J Nanobiotechnology. 2022;20:243. 35614462 10.1186/s12951-022-01444-3 PMC9131648

[15] Huang W, Li L, Liang Y, Yang Q, Mixdorf JC, Engle JW, ImmunoPET imaging of Nectin4 expression in gastric and bladder cancer using [64Cu]Cu-NOTA-Padcev. Mol Pharm. 2025. 10.1021/acs.molpharmaceut.5c00469 PMC12261917 40338113

[16] Kang L, Li C, Rosenkrans ZT, Engle JW, Wang R, Jiang D, Noninvasive evaluation of CD20 expression using 64Cu-Labeled F(ab’)2 fragments of Obinutuzumab in lymphoma. J Nucl Med. 2021;62:372–8. 32826320 10.2967/jnumed.120.246595 PMC8049347

[17] Ghani S, Deravi N, Pirzadeh M, Rafiee B, Gatabi ZR, Bandehpour M, Antibody fragment and targeted colorectal cancer therapy: a global systematic review. Curr Pharm Biotechnol. 2022;23:1061–71. 34375187 10.2174/1389201022666210810104226

[18] Huang W, Wang T, Qiu Y, Li C, Chen B, Song L, CD38-specific immunoPET imaging for multiple myeloma diagnosis and therapeutic monitoring: preclinical and first-in-human studies. Eur J Nucl Med Mol Imaging. 2025;52:1791–804. 39725695 10.1007/s00259-024-07036-7

[19] Huang W, Li L, Zhou Y, Yang Q, Mixdorf JC, Barnhart TE, Preclinical evaluation of zirconium-89 labeled anti-Trop2 antibody-drug conjugate (Trodelvy) for imaging in gastric cancer and triple-negative breast cancer. Eur J Nucl Med Mol Imaging. 2025. 10.1007/s00259-025-07106-4 PMC12119234 39878898

[20] Kang L, Li C, Yang Q, Sutherlin L, Wang L, Chen Z, 64Cu-labeled daratumumab F(ab’)2 fragment enables early visualization of CD38-positive lymphoma. Eur J Nucl Med Mol Imaging. 2022;49:1470–81. 34677626 10.1007/s00259-021-05593-9 PMC8940612

[21] Chakravarty R, Rohra N, Jadhav S, Sarma HD, Jain R, Chakraborty S. Biochemical separation of Cetuximab-Fab from papain-digested antibody fragments and radiolabeling with 64Cu for potential use in radioimmunotheranostics. Appl Radiat Isot. 2023;196:110795. 37004293 10.1016/j.apradiso.2023.110795

[22] Duan X, Xia L, Zhang Z, Ren Y, Pomper MG, Rowe SP, First-in-human study of the radioligand 68Ga-N188 targeting Nectin-4 for PET/CT imaging of advanced urothelial carcinoma. Clin Cancer Res. 2023;29:3395–407. 37093191 10.1158/1078-0432.CCR-23-0609

[23] Pavlasova G, Mraz M. The regulation and function of CD20: an “enigma” of B-cell biology and targeted therapy. Haematologica. 2020;105:1494–506. 32482755 10.3324/haematol.2019.243543 PMC7271567

[24] Suman SK, Kameswaran M, Pandey U, Sarma HD, Dash A. Preparation and preliminary bioevaluation studies of 68 Ga-NOTA-rituximab fragments as radioimmunoscintigraphic agents for non-Hodgkin lymphoma. J Labelled Comp Radiopharm. 2019;62:850–9. 31461549 10.1002/jlcr.3803

[25] Anz D, Mueller W, Golic M, Kunz WG, Rapp M, Koelzer VH, CD103 is a hallmark of tumor-infiltrating regulatory T cells. Int J Cancer. 2011;129:2417–26. 21207371 10.1002/ijc.25902

[26] Fan X, Ważyńska MA, Kol A, Perujo Holland N, Fernandes B, van Duijnhoven SMJ, Development of [89Zr]Zr-hCD103. Fab01A and [68Ga]Ga-hCD103.Fab01A for PET imaging to noninvasively assess cancer reactive T cell infiltration: fab-based CD103 immunoPET. EJNMMI Res. 2023;13:100. 37985555 10.1186/s13550-023-01043-9 PMC10661679

[27] Zettlitz KA, Tsai W-TK, Knowles SM, Salazar FB, Kobayashi N, Reiter RE, [89Zr]A2cDb immuno-PET of prostate cancer in a human prostate stem cell antigen knock-in (hPSCA KI) syngeneic model. Mol Imaging Biol. 2020;22:367–76. 31209779 10.1007/s11307-019-01386-7 PMC6920577

[28] Uehara T, Yokoyama M, Suzuki H, Hanaoka H, Arano Y. A gallium-67/68-labeled antibody fragment for Immuno-SPECT/PET shows low renal radioactivity without loss of tumor uptake. Clin Cancer Res. 2018;24:3309–16. 29666303 10.1158/1078-0432.CCR-18-0123

