# Supplementary material for: Rapid and specific immunoPET imaging of Nectin-4 in gastric cancer and non-small cell lung cancer using [64Cu]Cu-NOTA-EV-F(ab’)2
Source: Eur J Nucl Med Mol Imaging. 2025 Jun 21;53(1):619–32. doi: 10.1007/s00259-025-07402-z (PMC12261916; doi:10.1007/s00259-025-07402-z)
Supplement: Supplementary file 1 — (DOCX 2.01 MB) [file 259_2025_7402_MOESM1_ESM.docx]

**Supplementary** **materials and methods**

**Materials and methods**

**Preparation of F(ab')_2_ fragments**

EV was generously provided by the Department of Urology at Peking University First Hospital. F(ab')_2_ fragments of EV were prepared using the IdeS protease kit (Promega). The IdeS protease digestion process specifically cleaves the antibody below the hinge region, generating F(ab')_2_ fragments without the Fc domain. Consequently, the resulting F(ab')_2_ fragments are functionally identical to those derived from an unconjugated IgG.

EV (10 mg/mL) was incubated with IdeS protease in digestion buffer (50 mM sodium phosphate, 150 mM NaCl, pH 6.6) at 37°C for 1 h with moderate shaking (650 rpm) on a ThermoShaker (Biosan, USA). Following digestion, the sample was incubated with Magne® Protein A Beads^TM^ for 1 h on an end-over-end mixer (ThermoMixer system) to remove Fc fragments. The supernatant, containing purified F(ab')_2_ fragments was collected, and the concentration was determined using a NanoDrop spectrophotometer (Thermo Scientific) (EV-F(ab')_2_: 4.95 mg/mL). To ensure further purification, MagneHis^TM^ Ni Particles (Promega) was added to the digested sample, and the supernatant containing the desired F(ab')_2_ fragments was carefully collected using a pipette. The final products were analyzed using non-reducing Sodium Dodecyl Sulfate-Polyacrylamide Gel Electrophoresis (SDS-PAGE) and high-performance liquid chromatography (HPLC) to confirm purity and integrity.

**Conjugation and radiolabeling**

Conjugation of S-2-(4-isothiocyanatobenzyl)-1,4,7-triazacyclononane-1,4,7-triacetic acid (*p*-SCN-Bn-NOTA) (Macrocyclics, Plano, USA) was carried out at pH 9.0 for 2–3 h, using a NOTA-to-EV and NOTA-to-EV-F(ab')_2_ molar ratio of 15:1 (NOTA-EV: 5.53 mg/mL; NOTA-EV-F(ab')_2_: 4.47 mg/mL). ^64^Cu was produced using a PET trace cyclotron (GE Healthcare, Madison, WI) via the ^64^Ni(p,n)^64^Cu nuclear reaction.

For radiolabeling, approximately 74 MBq of ^64^Cu was diluted in 0.1 M sodium acetate buffer (pH 5.0) and incubated with NOTA-EV and NOTA-EV-F(ab')_2_ (~200 µg) at 37°C for 1 h. The resulting [^64^Cu]Cu-NOTA-EV and [^64^Cu]Cu-NOTA-EV-F(ab')_2_ conjugates were purified using a PD-10 column with PBS, and the radioactive fractions containing the [^64^Cu]Cu-labeled conjugates were collected. Labeling efficiency and radiochemical purity were evaluated using radio thin-layer chromatography (Radio-ITLC).

**Cell culture**

The human gastric cancer (NCI-N87, HGC-27) and lung cancer (H1975, H520) cell lines were obtained from the American Type Culture Collection (ATCC, Manassas, VA, USA). Cell lines were cultured in RPMI 1640 medium (Invitrogen; Gibco, USA), supplemented with 10% fetal bovine serum (FBS; HyClone, USA) and 5% penicillin/streptomycin (Gibco, USA). Cells were used for *in vitro* or *in vivo* experiments when they reached 70%–90% confluence.

**Animal model**

All animal experiments were conducted in compliance with protocols approved by the University of Wisconsin Institutional Animal Care and Use Committee (IACUC). For tumor model establishment, four- to five-week-old female Athymic Nude-Foxn1nu mice were purchased from Envigo (Indianapolis, IN). Tumors were generated by subcutaneous injection of 4–5 × 10^6^ cells, suspended in a 70 μL mixture of PBS and Matrigel (1:1, Corning, USA), into the front flank of each mouse. Tumor growth was monitored every other day, and mice were selected for *in vivo* experiments once tumor diameters reached 10–15 mm.

**Flow cytometry**

All cells were washed twice with cold PBS and adjusted to a final concentration of 1.0 × 10^5^ cells/mL before analysis using the Lightning cytometer (ThermoFisher Attune). Incubation was performed using EV, EV-F(ab')_2_, NOTA-EV, and NOTA-EV-F(ab')_2_ at a final concentration of 50 μg/mL for 1 h on ice in the dark. Subsequently, the cells were incubated with Alexa Fluor 488 (AF488)-labeled rabbit anti-human secondary antibodies at room temperature (RT) for 0.5 h. Data acquisition and analysis of mean fluorescence intensities were performed using FlowJo software.

**Cell uptake experiments**

Cells were seeded in 24-well plates at a density of 1.0 × 10^5^ cells per well, followed by the addition of 1 mL of serum-free medium containing 37 kBq of [^64^Cu]Cu-NOTA-EV and [^64^Cu]Cu-NOTA-EV-F(ab')_2_. The plates were incubated at 37°C for 2 h time points. After incubation, 1 mL of 1 M NaOH was added to each well for cell lysis, and the lysates were collected. The radioactivity in both the supernatants and lysates was measured using an automatic gamma counter (Wizard2, PerkinElmer).

**Cell binding assay**

For binding studies, NCI-N87 cells and H1975 cells were plated in a 96-well filter plate at a density of 1.0 × 10^5^ cells per well. A range of [^64^Cu]Cu-NOTA-EV and [^64^Cu]Cu-NOTA-EV-F(ab')_2_ solutions with final concentrations between 0.15 and 150 nM were prepared, and cells were incubated with these solutions at 37°C for 4 h. After incubation, the cells were thoroughly washed, and radioactivity was measured using a gamma counter. Surface Nectin-4 expression, apparent dissociation constant (*K_D_*), and maximum binding capacity (B_max_) were determined using total and nonspecific binding data analyzed with GraphPad Prism software, with the *K_D_* value largely depends on the surface density of Nectin-4 expression on the cell system which is used for affinity measurements. As EV and its derivatives are known to internalize upon Nectin-4 binding, the cell-associated radioactivity measured after a 4 h incubation at 37 °C likely reflects both membrane-bound and internalized fractions. Therefore, the calculated *K_D_* and B_max_ values represent apparent binding parameters under internalization-permissive conditions, rather than exclusive surface binding affinity.

**Immunofluorescent cell staining**

For the immunofluorescent staining assay, cells were plated in 35 mm confocal culture dishes at a density of 2.0 × 10^5^ cells and incubated overnight. Following fixation with 3% paraformaldehyde at 4°C, the cells were treated with EV (1:800), washed three times with cold PBS, and subsequently incubated with Alexa Fluor 488-labeled rabbit anti-mouse secondary antibodies at RT for 30 minutes. After three additional washes with cold PBS, the cells were stained using a DAPI-containing mounting medium. Coverslips were then placed, and imaging was conducted using a Nikon A1R confocal laser scanning microscope (Nikon, Inc., Melville, NY).

**ImmunoPET imaging**

PET imaging studies were conducted using an Inveon microPET scanner designed for rodents (Siemens Medical Solutions USA, Inc.). Tumor-bearing mice received an intravenous injection of 7.4–11.1 MBq of [^64^Cu]Cu-NOTA-EV and [^64^Cu]Cu-NOTA-EV-F(ab')_2_, and imaging was performed at 1, 4, 12, 24, and 48 h post-injection (p.i.). To improve image quality, 30 million coincidence events per mouse were acquired for every static PET emission scan (acquired over a 20-minute duration per scan). Images were calibrated to display signals within a range of 0–10% of the injected dose per volume, expressed as %ID/g. For blocking studies, mice were injected with an excess of unlabeled EV (2 mg) 2 h before the experiment. Quantitative analysis involved delineating regions of interest (ROI) in the heart, liver, kidneys, muscles, spleen, and tumors. Tumor-to-heart (T/H) and tumor-to-muscle (T/M) ratios were subsequently calculated to assess tracer distribution.

***Ex vivo* biodistribution**

After the final imaging session, mice were euthanized via carbon dioxide asphyxiation. Key organs and tissues, including the tumor, heart, liver, spleen, lungs, kidneys, stomach, intestines, tail, muscles, bones, brain, skin, and blood, were excised and weighed. The radioactivity of each sample was quantified using an automated gamma counter, and biodistribution was expressed as %ID/g.

**Radiation dosimetry prediction**

Balb/c mice (female, 4–5 weeks old) were divided into 4 groups (n = 3). Balb/c mice were then injected with 0.74 MBq of [^64^Cu]Cu-NOTA-EV and [^64^Cu]Cu-NOTA-EV-F(ab')_2_ (100 μL) through the tail vein and sacrificed 1, 4, 24 and 48 h after injection. The extrapolated doses to adult females were calculated using decay corrected ^64^Cu biodistribution data. The dosimetry analysis was performed using the OLINDA/EXM dose-spherical model provided by the OLINDA/EXM software. Time-activity curves were fitted using monoexponential decay models based on organ-specific %ID/g values collected at 1, 4, 24, and 48 h post-injection. These curves were scaled to human organ masses using established allometric scaling principles. While this method is widely accepted for first-in-human dosimetry prediction and is commonly used in preclinical radiopharmaceutical studies, it does have inherent limitations. Notably, interspecies differences in metabolism, clearance pathways, and organ size may introduce variability in dose estimates. Furthermore, organ-specific radiotracer retention observed in mice—particularly in the kidneys and liver—may not fully represent human biodistribution profiles.

**Histological analysis**

Histological assessments included hematoxylin-eosin (H&E) staining, immunohistochemistry (IHC), and immunofluorescence. Immunofluorescent staining was conducted on tumor tissues and major organs, including the heart, lungs, liver, spleen, stomach, pancreas, intestine, and kidneys, to evaluate Nectin-4 expression according to established protocols. Primary antibodies used were anti-human Nectin-4 (1:300, Abcam) and mouse anti-human CD31 (1:300, Servicebio). Secondary antibodies consisted of AF488-labeled goat anti-rabbit IgG (1:200, Servicebio) and Cy3-labeled goat anti-mouse IgG (1:200, Servicebio). Tissue morphology and staining patterns were analyzed and captured using a NIKON Eclipse Ti confocal microscope.

**Statistical analysis**

Quantitative data are expressed as mean ± standard deviation (SD). Statistical analyses were conducted using the *Student's* *t-test* or *one- or two-way ANOVA* in GraphPad Prism version 8.0. A P-value < 0.05 was considered statistically significant. All experiments were performed in triplicate or more, with results presented as mean ± SD unless specified otherwise. Statistical significance in the figures is denoted by asterisks: * *P* < 0.05; ** *P* < 0.01; *** *P* < 0.001; **** *P* < 0.0001.

**Supplementary results**

**Radiosynthesis of [^64^Cu]Cu-NOTA-EV-F(ab')_2_**

Radiolabeling with ^64^CuCl_2_ at pH 5.0 and 37°C for 1 h resulted in a high radiochemical yield (85.40 ± 2.43, n = 5), as verified by radio-ITLC (**Supplementary Figure 1A**). Following purification with PD-10 columns, the radiochemical purity (RCP) of the tracers surpassed 99%, with no detectable free ^64^Cu in the target fractions of [^64^Cu]Cu-NOTA-EV-F(ab')_2_ **(Supplementary Figure 1B**). *In vitro* stability studies confirmed that [^64^Cu]Cu-NOTA-EV-F(ab')_2_ maintained a high RCP (> 98%) after 24 h of incubation in 0.01 M PBS and 5% human serum albumin (HSA), demonstrating excellent stability and suitability for further research applications **(Supplementary Figure 1C and 1D**).


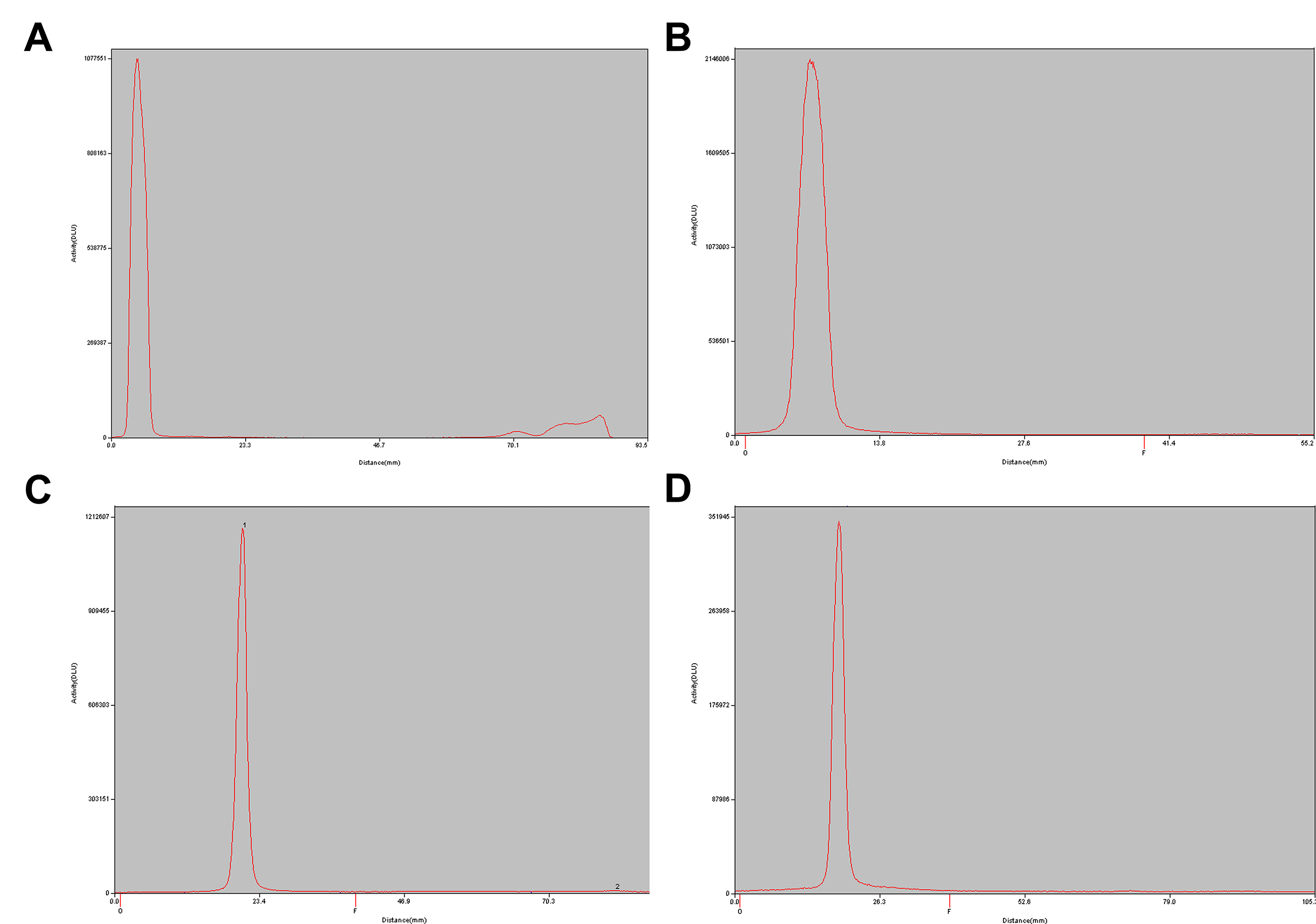


**Figure S1. Radio-ITLC analysis of the radioactive assay utilizing [^64^Cu]Cu-NOTA-EV-F(ab')_2_ as the substrate. (A)** Radiochemical yield of [^64^Cu]Cu-NOTA-EV-F(ab')_2_. **(B)** Radiochemical purity of [^64^Cu]Cu-NOTA-EV-F(ab')_2_ following purification with a PD-10 column. **(C–D)** Radiochemical purity of [^64^Cu]Cu-NOTA-EV-F(ab')_2_ after 24 h of incubation in 0.01 M PBS and 5% human serum albumin.


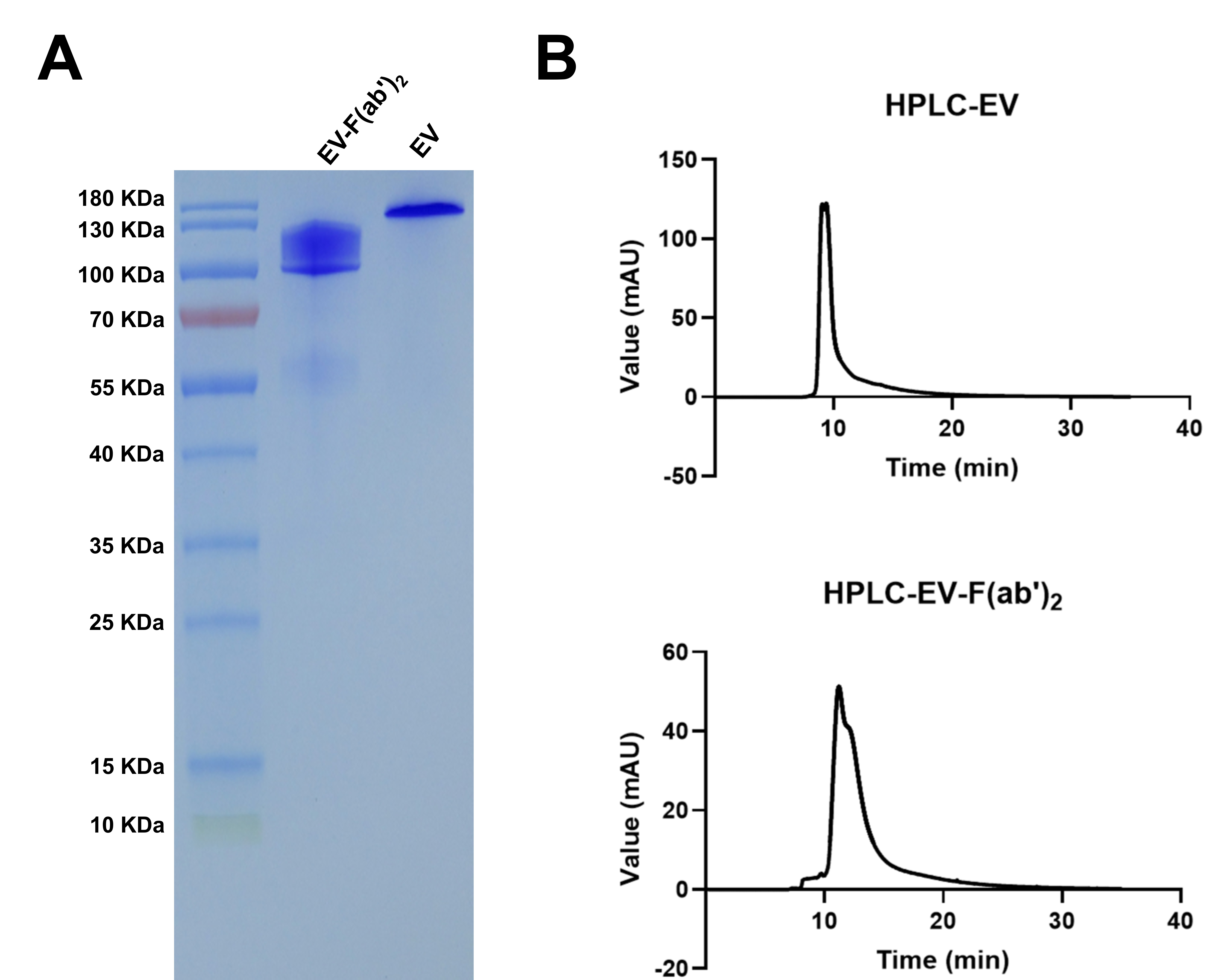


**Figure S2.** **Characterization of EV-F(ab')_2_. (A)** SDS-PAGE analysis showing characteristic bands for EV and EV-F(ab')_2_ at approximately 150 kDa and 100 kDa, respectively. **(B)** HPLC elution profile with the main peak for EV-F(ab')_2_ at approximately 1.5 min following the EV peak.

**Radiation dosimetry extrapolation to humans**

Dosimetry analysis was conducted using OLINDA/EXM software. Estimated human dosimetry was derived from average %ID/g values obtained from serial PET scans on BALB/c mice, converted to %ID in humans. Assuming similar biodistribution in adult humans as in animal models, a monoexponential model was applied to the time–activity curves. OLINDA provides effective dose outputs, and weighting factors from the International Commission on Radiological Protection Publication 103 were utilized to convert these to absorbed doses for each organ. The estimated radiation doses of [^64^Cu]Cu-NOTA-EV-F(ab')_2_ for human organs, derived from biodistribution data, are summarized in **Table 1**. Similarly, the radiation dose estimates for [^64^Cu]Cu-NOTA-EV are provided in **Table S1**. The estimated systemic effective dose for an adult woman was 0.0353 mSv/MBq, which falls within acceptable limits for conventional nuclear medicine research. The predictions should be regarded as preliminary estimates requiring further validation in clinical settings.

**Table S1.** Human organ radiation dosimetry estimation of [^64^Cu]Cu-NOTA-EV

| Target Organ | mSv/MBq |
| --- | --- |
| Adrenals | 3.26E-04 |
| Brain | 7.40E-05 |
| Breasts | 3.43E-03 |
| Esophagus | 1.33E-03 |
| Eyes | 0.00E00 |
| Gallbladder Wall | 3.15E-04 |
| Left colon | 1.71E-03 |
| Small Intestine | 3.36E-04 |
| Stomach Wall | 4.31E-03 |
| Right colon | 1.65E-03 |
| Rectum | 7.79E-04 |
| Heart Wall | 9.28E-04 |
| Kidneys | 3.40E-04 |
| Liver | 1.98E-03 |
| Lungs | 5.52E-03 |
| Ovaries | 1.36E-03 |
| Pancreas | 2.07E-04 |
| Salivary Glands | 2.90E-04 |
| Red Marrow | 3.22E-03 |
| Osteogenic Cells | 2.55E-04 |
| Spleen | 3.47E-04 |
| Thymus | 3.24E-04 |
| Thyroid | 1.22E-03 |
| Urinary Bladder Wall | 1.21E-03 |
| Uterus | 1.57E-04 |
| Effective Dose | 3.16E-02 |

**Table S2.** *Ex vivo* biodistribution data of [^64^Cu]Cu-NOTA-EV and [^64^Cu]Cu-NOTA-EV-F(ab')_2_ in gastric cancer mouse models at 48 h post-injection

|  | [^64^Cu]Cu-NOTA-EV in NCI-N87 | [^64^Cu]Cu-NOTA-EV-F(ab')_2_ in NCI-N87 | [^64^Cu]Cu-NOTA-EV-F(ab')_2_ in HGC-27 | [^64^Cu]Cu-NOTA-EV-F(ab')_2_ blocking in NCI-N87 |
| --- | --- | --- | --- | --- |
| Blood | 10.52 ± 1.44 | 1.39 ± 0.13 | 1.16 ± 0.20 | 1.22 ± 0.09 |
| Skin | 2.27 ± 0.49 | 0.79 ± 0.29 | 0.87 ± 0.23 | 1.08 ± 0.27 |
| Muscle | 1.12 ± 0.13 | 0.29 ± 0.10 | 0.34 ± 0.06 | 0.35 ± 0.04 |
| Bone | 0.91 ± 0.20 | 0.40 ± 0.12 | 0.32 ± 0.06 | 0.41 ± 0.07 |
| Heart | 3.73 ± 1.10 | 0.72 ± 0.05 | 0.70 ± 0.06 | 0.85 ± 0.11 |
| Lung | 4.60 ± 1.10 | 0.86 ± 0.08 | 0.99 ± 0.14 | 1.00 ± 0.10 |
| Liver | 6.06 ± 1.43 | 3.31 ± 0.46 | 3.10 ± 0.21 | 3.52 ± 0.34 |
| Kidney | 4.27 ± 0.29 | 22.10 ± 3.14 | 21.20 ± 2.10 | 25.57 ± 2.59 |
| Spleen | 5.13 ± 0.53 | 0.81 ± 0.11 | 0.81 ± 0.16 | 0.79 ± 0.17 |
| Pancreas | 2.21 ± 0.52 | 0.36 ± 0.02 | 0.44 ± 0.00 | 0.67 ± 0.36 |
| Stomach | 1.97 ± 0.46 | 0.58 ± 0.04 | 0.47 ± 0.07 | 0.51 ± 0.10 |
| Intestine | 1.77 ± 0.12 | 0.59 ± 0.06 | 0.58 ± 0.04 | 0.65 ± 0.13 |
| Tail | 2.35 ± 0.17 | 0.73 ± 0.36 | 0.94 ± 0.20 | 0.78 ± 0.23 |
| Brain | 0.62 ± 0.18 | 0.08 ± 0.01 | 0.07 ± 0.04 | 0.08 ± 0.01 |
| Tumor | 14.00 ± 1.50 | 5.81 ± 0.83 | 1.37 ± 0.20 | 2.68 ± 0.59 |

Note: n = 3 per group; data presented as %ID/g, mean ± SD.

**Table S3.** *Ex vivo* biodistribution data of [^64^Cu]Cu-NOTA-EV and [^64^Cu]Cu-NOTA-EV-F(ab')_2_ in non-small cell lung cancer mouse models at 48 h post-injection

|  | [^64^Cu]Cu-NOTA-EV in H1975 | [^64^Cu]Cu-NOTA-EV-F(ab')_2_ in H1975 | [^64^Cu]Cu-NOTA-EV-F(ab')_2_ in H520 | [^64^Cu]Cu-NOTA-EV-F(ab')_2_ blocking in H1975 |
| --- | --- | --- | --- | --- |
| Blood | 10.63 ± 1.43 | 1.58 ± 0.53 | 1.32 ± 0.20 | 1.23 ± 0.13 |
| Skin | 1.26 ± 0.20 | 0.85 ± 0.42 | 0.76 ± 0.08 | 0.92 ± 0.14 |
| Muscle | 0.66 ± 0.06 | 0.28 ± 0.06 | 0.30 ± 0.03 | 0.35 ± 0.05 |
| Bone | 0.99 ± 0.15 | 0.30 ± 0.05 | 0.39 ± 0.05 | 0.45 ± 0.06 |
| Heart | 3.50 ± 0.61 | 0.71 ± 0.06 | 0.73 ± 0.08 | 0.77 ± 0.04 |
| Lung | 4.86 ± 0.49 | 0.86 ± 0.09 | 0.94 ± 0.05 | 1.02 ± 0.06 |
| Liver | 4.67 ± 0.26 | 2.88 ± 0.43 | 3.63 ± 0.69 | 3.36 ± 0.10 |
| Kidney | 3.60 ± 0.11 | 14.26 ± 2.63 | 20.20 ± 2.91 | 15.83 ± 2.73 |
| Spleen | 3.38 ± 1.19 | 0.69 ± 0.06 | 0.99 ± 0.06 | 0.88 ± 0.09 |
| Pancreas | 1.55 ± 0.13 | 0.30 ± 0.06 | 0.39 ± 0.03 | 0.35 ± 0.05 |
| Stomach | 1.37 ± 0.13 | 0.59 ± 0.11 | 0.61 ± 0.04 | 0.46 ± 0.11 |
| Intestine | 1.01 ± 0.24 | 0.51 ± 0.07 | 0.55 ± 0.09 | 0.59 ± 0.06 |
| Tail | 2.20 ± 0.64 | 0.77 ± 0.50 | 0.69 ± 0.11 | 0.80 ± 0.12 |
| Brain | 0.34 ± 0.12 | 0.073 ± 0.003 | 0.078 ± 0.008 | 0.088 ± 0.005 |
| Tumor | 12.50 ± 2.26 | 5.45 ± 0.63 | 1.41 ± 0.08 | 3.10 ± 0.26 |

Note: n = 3 per group; data presented as %ID/g, mean ± SD.
